# Discovery of a highly selective JAK3 inhibitor for the treatment of rheumatoid arthritis

**DOI:** 10.1038/s41598-018-23569-y

**Published:** 2018-03-27

**Authors:** Heying Pei, Linhong He, Mingfeng Shao, Zhuang Yang, Yan Ran, Dan Li, Yuanyuan Zhou, Minghai Tang, Taijin Wang, Yanqiu Gong, Xiaoxin Chen, Shengyong Yang, Mingli Xiang, Lijuan Chen

**Affiliations:** 1 State Key Laboratory of Biotherapy/Collaborative Innovation Center of Biotherapy and Cancer Center, West China Hospital of Sichuan University, Chengdu, China; 2 Guangdong Zhongsheng Pharmaceutical Co., Ltd., Dongguan, Guangdong 523325 China

## Abstract

Janus tyrosine kinase 3 (JAK3) is expressed in lymphoid cells and is involved in the signalling of T cell functions. The development of a selective JAK3 inhibitor has been shown to have a potential benefit in the treatment of autoimmune disorders. In this article, we developed the 4-aminopiperidine-based compound **RB1**, which was highly selective for JAK3 inhibition, with an IC_50_ of value of 40 nM, but did not inhibit JAK1, JAK2 or tyrosine kinase 2 (TYK2) at concentrations up to 5 µM. Furthermore, **RB1** also exhibited favourable selectivity against a panel of representative kinases. In a battery of cytokine-stimulated cell-based assays, this potent inhibitor of JAK3 activity with good selectivity against other kinases could potently inhibit JAK3 activity over the activity of JAK1 or JAK2 (over at least 100-fold). A combination of liquid chromatography-mass spectrometry (LC-MS) experiments validated that **RB1** covalently modified the unique cysteine 909 residue in JAK3. *In vivo*, **RB1** exerted significantly improved pathology in the joints of a collagen-induced arthritis mouse model. The reasonable pharmacokinetics properties (F = 72.52%, T1/2 = 14.6 h) and favourable results of toxicology experiments (LD_50_ > 2 g/kg) suggest that **RB1** has the potential to be an efficacious treatment for RA.

## Introduction

Rheumatoid arthritis (RA) is a chronic, systemic disease characterized by persistent inflammatory synovitis that typically involves peripheral joints in a symmetric distribution^1^. Inflammatory cytokines, such as tumor necrosis factor α (TNF-α), IL-1, and IL-6, play important roles in the pathogenesis of RA. For such cytokines to exert their biologic activities, the appropriate intracellular signalling pathways must be activated via their specific receptors on the cell surface. After receptor binding in a cytokine response, tyrosine kinases are the first intracellular signalling molecules to be activated and recruited at the sites of inflammation^2,3^. Therefore, recent studies have focused on tyrosine kinases as potential targets for the treatment of RA. Among them, inhibitors specific for the JAK family are, so far, the most efficacious in treating RA.

JAKs are a family of nonreceptor protein tyrosine kinases that are critical for cytokine-receptor-binding-triggered signal transduction through STAT to the nuclei of cells. The JAK family consists of four members: JAK1, JAK2, JAK3 and TYK2^4^. In mammals JAK1, JAK2 and TYK2 are ubiquitously expressed. In contrast, the expression of JAK3 is more restricted; it is predominantly expressed in haematopoietic cells and is highly regulated with cell development and activation^5,6^. JAK3 is solely activated by type I cytokine receptors featuring a common γ-chain (γc) subunit that are activated by IL-2, IL-4, IL-7, IL-9, IL-15, and IL-21^7^; mutations in either the γ-chain or JAK3 have been identified in humans as a cause of severe combined immunodeficiency disease (SCID), which manifests as a depletion of T, B, and natural killer (NK) cells with no other defects^7,8^. In addition, it has been reported that the inhibition of JAK2 can lead to dose-limiting side effects including anaemia and neutropenia^9^. These observations suggest that selective JAK3 inhibition should be sufficient for immunosuppression without causing effects outside the immune system; thus, JAK3 has been identified as a potential target to treat RA^8,10,11^. In 2012, the U.S. FDA has approved Tofacitinib as an oral JAK3 inhibitor to treat adults with moderately to severely active RA.

**Figure 1:**
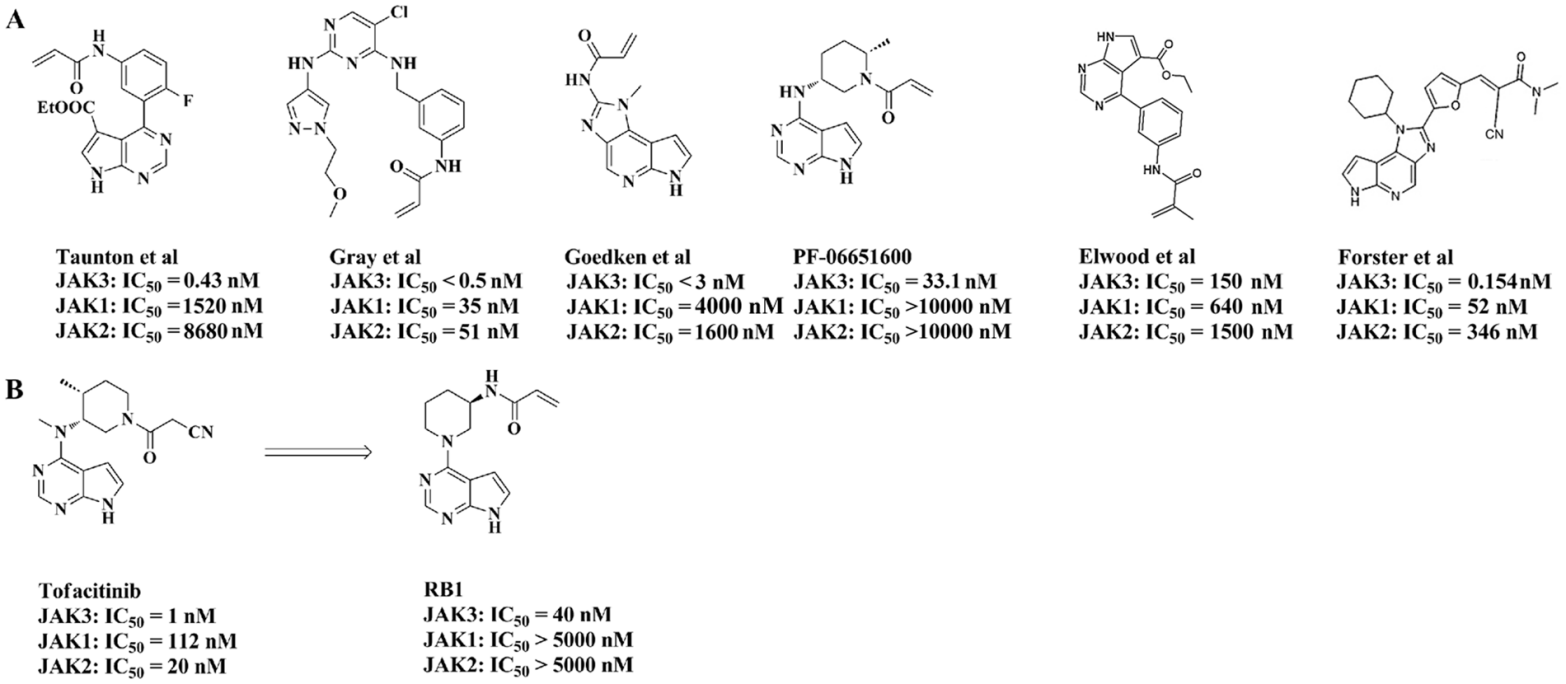
**RB1**, a JAK3 selective inhibitor. (**A**) Structure of JAK3 covalent inhibitors. (**B**) Structure of Tofacitinib and **RB1**. IC_50_ values were determined at the K_m_ for JAKs and are the mean of at least three experiments.

Tofacitinib is a first-in-class JAK3 inhibitor with low nanomolar inhibition for JAK3, but it still shows limited selectivity against JAK1 and JAK2 (JAK3: IC_50_ = 1 nM; JAK1: IC_50_ = 112 nM; JAK2: IC_50_ = 20 nM)^7,12^. Although Tofacitinib provides a good treatment for RA, it also causes several severe side effects, such as anaemia and neutropenia, which are possibly caused by the inhibition of JAK2^12–14^; recent IC_50_ data highlight the fact that Tofacitinib is indeed not an isoform-selective JAK3 inhibitor^15–20^, which means Tofacitinib is currently considered and widely accepted as a pan-JAK inhibitor. Therefore, a selective JAK3 inhibitor has the potential benefit of alleviating undesirable side effects and would be safe for the treatment of RA that requires long-term therapy^10^. Due to the high homology in the active sites of the JAKs, the development of a highly selective JAK3 small-molecule inhibitor remains challenging^21,22^. To date, there is only a single isoform-selective JAK3 inhibitor, PF-06651600, under a phase 2 clinical evaluation to treat RA. This compound inhibits JAK3 kinase activity with an IC_50_ of 33.1 nM but without activity (IC_50_ > 10000 nM) against JAK1, JAK2, or TYK2^23,24^. Analysis of the structures of JAK family members indicates that JAK3 is unique in having a cysteine residue at the binding pocket^22–24^. This residue is Cys909, that can be used to confer selectivity by targeting the formation of a covalent interaction^22–24^. By employing this mechanism, several highly selective JAK3 inhibitors have been reported and are listed in Fig. 1A^22,24–28^. Encouraged by these results, we decided to optimize the structure of Tofacitinib in an attempt to develop a highly selective JAK3 inhibitor, which led to the discovery of the compound **RB1** (Fig. 1B). In this paper, we describe **RB1**, a newly discovered irreversible covalent JAK3 inhibitor that exhibits higher JAK isoform selectivity for JAK3 and shows good pharmacokinetics properties, similar to the compound PF-65501600. Moreover, **RB1** significantly improves arthritic symptoms in CIA mice without any side effects.

## Results

### Chemistry

Based on Tofacitinib, a series of irreversibly covalent JAK3-selective inhibitors were designed and synthesized. This strategy used 4-aminopiperidine to replaced the (3 S, 4 S)-4-methylpiperidin-3-yl portion of Tofacitinib, which was connected with 6-chloropurine, and then several electrophilic “warheads” were coupled with the primary amino group. The JAK3 kinase activity and selectivity were evaluated *in vitro*, and the **RB1** compound showed the most potent activity (these studies will be published soon). The details of the design and synthesis methods are shown in Supplementary Fig. S1, and the 1H-NMR and 13C-NMR data are also presented in Supplementary Fig. S2.

### RB1 selectively inhibits JAK3

In the presence of an ATP concentration at K_m_ for ATP for each JAK isoform, **RB1** (Fig. 1B) inhibited JAK3 kinase activity with an IC_50_ value of 40 nM without inhibiting JAK1, JAK2 or TYK2 (IC_50_ > 5000 nM) (Table 1). As kinases that share a cysteine at the structurally equivalent position may be non-selectively targeted by covalent kinase inhibitors, we further assessed the selectivity of **RB1** against 10 protein kinases that contain a cysteine in the equivalent position as that of JAK3, including the TEC family (BMX, BTK, ITK, TXK, and TEC), the ErbB family (EGFR, ERBB4, and ERBB2), JAK3 and BLK^29^. **RB1** exhibited excellent selectivity among this conserved cysteine panel, with a high inhibition (99.1%) of JAK3 and a low inhibition of other kinases, less than 15% at 1 µM (Table 2). Furthermore, we determined the selectivity of **RB1** by measuring the binding affinities of **RB1** to a panel of 81 representative kinases. **RB1** was screened against the kinase set at a fixed concentration of 1 μM, and all screening data are represented by a % activity value. The results, shown in Supplementary Table S1, indicated that **RB1** exhibited excellent overall selectivity for JAK3. **RB1** only showed the highest binding affinity to JAK3, with a 0.9% residual kinase activity at a 1 µM concentration, and showed no inhibition of the other 75 representative kinases, with the exception of Aurora-A, Aurora-B, CLK2, MKK7β and PKG1α. Although **RB1** exhibited a weak inhibition of these five kinases, their estimated IC_50_ values were all over 800 nM. The calculated selectivity scores of S(1), S(10), and S(35), whose definitions are given in the Methods^30^, were 0.012, 0.012, and 0.086, respectively. These data indicated that **RB1** could potently inhibit JAK3 while retaining a high level of kinase panel selectivity.

**Table 1:** RB1 Inhibition of JAK Isoforms in Biochemical Assays^*a*^.

JAK isoform	ATP [*μ*M]	IC_50_ [nM]	SEM (n)
JAK1	90	>5000	(2)
JAK2	20	>5000	(2)
JAK3	6.2	40	2.2(2)
TYK2	16.0	>5000	(2)

^a^Enzymatic assays were performed in the presence of ATP concentrations at K_m_ μM to each JAK isoform. IC_50_ values represent the geometric mean of “n” independent experiments. SEM means the standard error of the mean. (n) Indicates the number of independent experiments.

**Table 2:** Selectivity among Cysteine family members^*a*^.

kinase	% Inhibition (1 µM)									
	JAK3	BMX	BTK	ITK	TXK	TEC	BLK	EGFR	ERBB4	ERBB2
**RB1**	99.1	4	−0.95	−5.9	7.25	14	8.35	11.25	−3.5	2.85

^a^All percent inhibition data are the mean of at least 2 independent measurements, with calculated standard errors below 15%.

### RB1 inhibits JAK3 selectively in cell-based assays

In addition to enzymatic selectivity, functional selectivity was characterized in human peripheral blood mononuclear cells (PBMCs), which consists of various populations of immune cells that can be stimulated by different cytokines. For example, IL-2-induced STAT5 phosphorylation is mediated by JAK3 and JAK1^31^, IL-6-induced STAT3 phosphorylation is thought to be triggered by JAK1, JAK2, and TYK2^32^, and GM-CSF-induced STAT5 phosphorylation is dependent on JAK2^33^. Thus, the potency and selectivity of **RB1** and Tofacitinib in a cellular context were assessed in total lymphocytes in hPBMCs by flow cytometry. As shown in Table 3 and Supplementary Fig. S3, **RB1** and Tofacitinib inhibited the phosphorylation of STAT5 elicited by IL-2 at IC_50_ values of 105 nM and 31 nM, respectively. Meanwhile, Tofacitinib also inhibited the IL-6-induced phosphorylation of STAT3 and the GM-CSF-induced phosphorylation of STAT5 at IC_50_ values of 73 nM and 659 nM, respectively, and showed only a 2.4-fold and a 22-fold selectivity over the IL-2 stimulated pathway. Although **RB1** showed relatively lower inhibition of the phosphorylation of STAT5 elicited by IL-2 compared to that of Tofacitinib, it showed 400-fold selectivity over the IL-2 stimulated pathway. These observations suggested that **RB1** exhibits higher JAK isoform selectivity for JAK3 in comparison to Tofacitinib (Table 3).

**Table 3:** Cellular Potency of RB1 in Total Lymphocytes in Human Peripheral Blood Mononuclear Cells^*a*^.

JAKs Involved	Trigger	Readout	IC_50_^44^		Selectivity	
					JAK1/JAK3 or JAK2/JAK3	
			**RB1**	Tofacitinib	**RB1**	Tofacitinib
JAK1/JAK3	IL-2	pSTAT5	105	30	—	—
JAK1	IL-6	pSTAT3	>40000	73	>400	2.4
JAK2	GM-CSF	pSTAT5	>10000	659	>100	22

^a^**RB1** cellular IC_50_ values for the phosphorylation of STAT proteins in response to various cytokine treatments.

**Figure 2:**
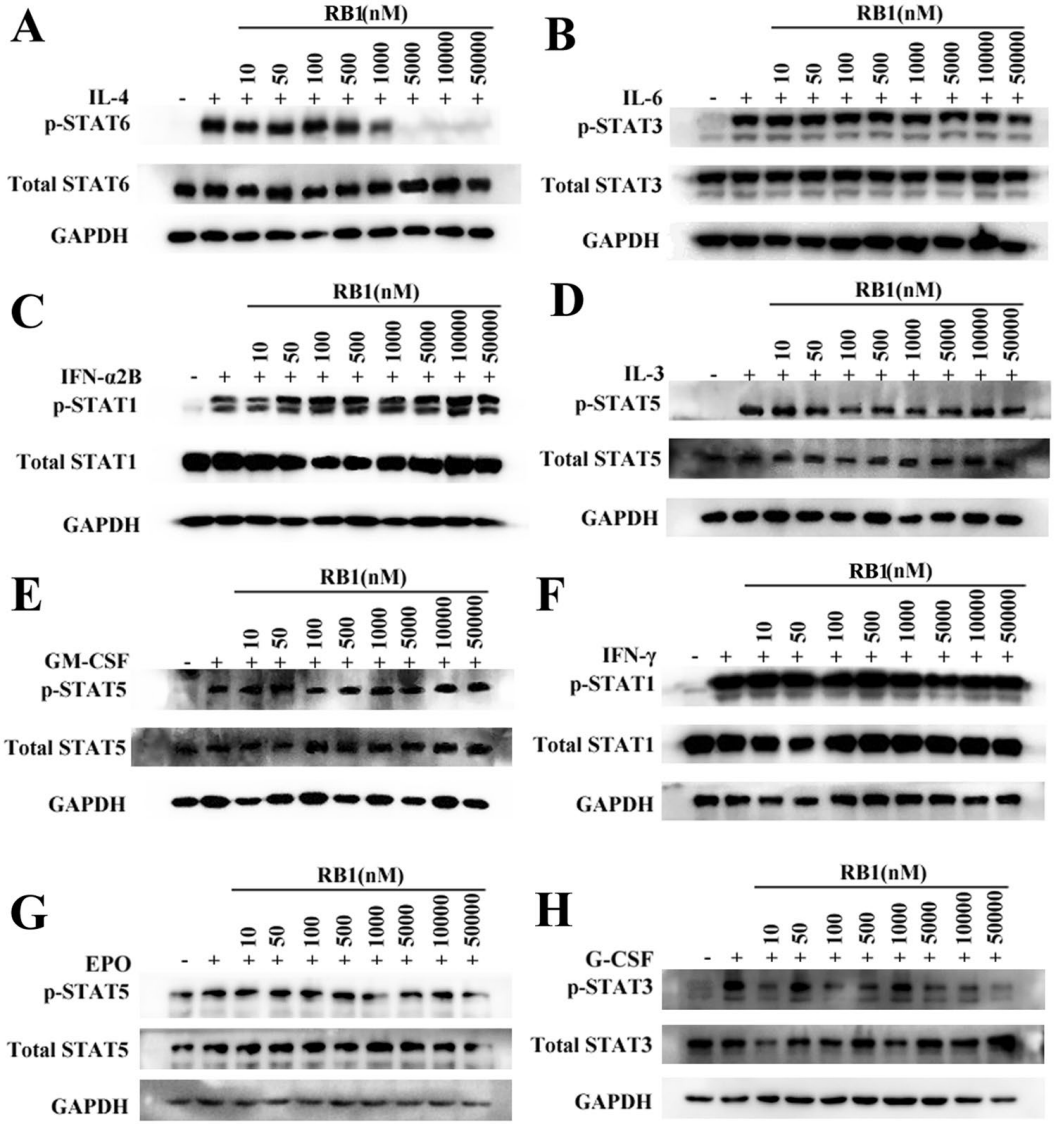
Identification of **RB1** as a highly selective JAK3 inhibitor. Cells were pretreated with **RB1** for 1 h, followed by treatment with IL-4, IL-6, IFN-α2b, IL-3, GM-CSF, IFN-γ, EPO or G-CSF for an additional 20 to 60 minutes. Cells were lysed with sample buffer, and the lysates were analysed using immunoblotting. (**A**) Western blot analysis of STAT6 phosphorylation after treatment with **RB1** in THP-1 cell lines. (**B**) Western blot analysis of STAT3 phosphorylation after treatment with **RB1** in TF-1 cell lines. (**C**) Western blot analysis of STAT1 phosphorylation after treatment with **RB1** in U2OS cell lines. (**D**) Western blot analysis of STAT5 phosphorylation after treatment with **RB1** in TF-1 cell lines. (**E**) Western blot analysis of STAT5 phosphorylation after treatment with **RB1** in TF-1 cell lines. (**F**) Western blot analysis of STAT1 phosphorylation after treatment with **RB1** in U2OS cell lines. (**G**) Western blot analysis of STAT5 phosphorylation after treatment with **RB1** in HEL cell lines. (**H**) Western blot analysis of STAT3 phosphorylation after treatment with **RB1** in HEL cell lines. Each value represents the average of 2 independent experiments, where each experiment consisted of two replicates. Uncropped images of blots are shown in Supplementary Fig. S10.

To further determine the selectivity of **RB1** for the inhibition of different JAK isotypes within cells, we used a battery of cytokine-stimulated cell-based assays. Cell lines were preincubated with **RB1** and treated with cytokines that employ different JAK heterodimeric or JAK3 homodimeric complexes for signalling (Fig. 2, Supplementary Table S2). All results were measured by Western blot and quantified by greyscale intensity levels. To assess JAK1 and JAK3 signalling, we stimulated THP-1 cells via IL-4 and measured the levels of phosphorylated STAT6. In this system, **RB1** potently and concentration-dependently inhibited the phosphorylation of JAK3 with an IC_50_ value of 53.1 nM (Supplementary Table S2 and Supplementary Fig. S4A) and STAT6 (Fig. 2A). Compared with a JAK1 obligatory assay (IL-6 phosphorylation of STAT3 in TF-1 cells and IFN-α2b phosphorylation of STAT1 in U2OS cells) (Fig. 2B,C and Supplementary Fig. S4B, C), **RB1** showed a very weak ability to inhibit STAT and JAK phosphorylation, with IC_50_ values over 50 μM. Similarly, in IL-3- and GM-CSF-mediated phosphorylation of STAT5 in TF-1 cells, and in EPO-mediated phosphorylation of STAT5 in HEL cells, which requires the activity of JAK2, the IC_50_ values for **RB1** could not be determined accurately (>50 μM) (Fig. 2D,E,G and Supplementary Fig. S4D,E,G). In addition, IFN-γ-induced phosphorylation of STAT1 in U2OS cells and G-CSF-induced phosphorylation of STAT3 in HEL cells, which involve JAK1/JAK2 signalling, were not affected, even when exposed to **RB1** at concentrations over 50 μM (Fig. 2F,H and Supplementary Fig. S4F,H). These data in the enzyme and cell-based assays *in vitro* indicate that **RB1** inhibits the JAK3 isoform rather than JAK1 or JAK2.

### RB1 showed irreversible inhibition of JAK3

**Figure 3:**
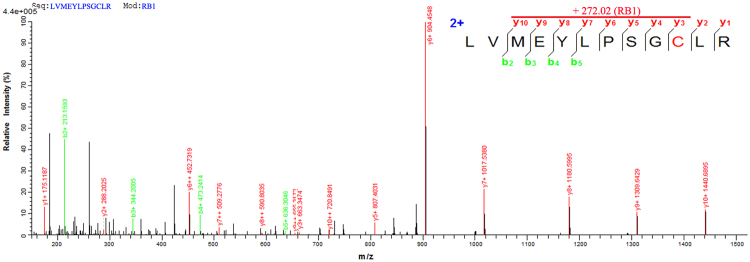
**RB1** showed the irreversible inhibition of JAK3. Mass spectrometry mapping shows that Cys909 is modified by **RB1**. The MS/MS spectrum of peptide LVMEYLPSGCLR depicts the modification of Cys-909 by **RB1** (marked by red colour).

The high selectivity of **RB1** in enzyme and cell-based assays for the inhibition of JAK3 over the other three JAK isoforms may have been achieved by the irreversible covalent binding to Cys909 in JAK3, which is a Ser residue in the other three JAKs. We incubated JAK3 with **RB1**, and the resulting mixture was subjected to LC-MS/MS analysis. The m/z of the Cys909-containing peptide (900-LVMEYLPSGCLR-911) had a mass increase of 272.02 Da, which was consistent with the calculated molecular weight for the addition of **RB1** to this peptide. Further fragmentation of this peptide produced a series of b/y-ion fragments. According to the MS/MS spectrum analysis, experiments with **RB1** showed the expected change in intact molecular weight upon the covalent modification of the intact JAK3 following the loss of the H_2_O leaving group (253.02 Da), suggesting Cys909 was the covalently modified residue (Fig. 3). In summary, our biochemical data indicate that **RB1** covalently modify JAK3 in an apparently irreversible manner. A jump dilution experiment also confirmed that **RB1** was an irreversible JAK3 inhibitor, because no active JAK3 was detected over time after preincubation with **RB1** (Supplementary Fig. S5).

### RB1 has suitable pharmacokinetics properties and low toxicity in pharmacodynamics evaluations

We evaluated the pharmacokinetic properties of **RB1** in Sprague Dawley (SD) rats following separate intravenous and oral administration. As we expected, **RB1** was rapidly absorbed (Tmax = 0.25 h) following a 10 mg/kg oral doses. Importantly, **RB1** exhibited favourable oral bioavailability (72.5%) and a suitable half-time (14.6 h) (Supplementary Table S3). To investigate whether **RB1** was safe for oral administration, acute toxicity studies were completed to evaluate the safety of **RB1** in SD rats. A series of doses of the compound (from 0.4 g/kg to 2.0 g/kg) were independently administered orally to rats. Careful observations showed that **RB1** exhibited no observable adverse effects on the body weight, behaviour, or appetite of the tested rats. In addition, we administered single doses of **RB1** and performed an acute oral toxicity assay in BALB/c mice by observing adverse effects within a 14-days recovery time. After careful observation, **RB1** exhibited no obvious adverse results in mice; they were active and healthy, with no signs of toxicity, adverse pharmacological effects or abnormal behaviours. Haematology analyses were also assessed after 14 days. As expected, the oral administration of a high dose of **RB1** did not induce significant acute haematological toxicity in WBC and lymphocyte counts even at a 1000 mg/kg dose in the **RB1-**treated group, which showed no adverse effects on lymphocyte development (Supplementary Table S4). Based on the favourable pharmacokinetics properties and low toxicity of **RB1**, we were encouraged to further evaluate the efficacy of **RB1** in a collagen-induced arthritis model *in vivo*.

### RB1 is efficacious in a mouse collagen-induced arthritis model

**Figure 4:**
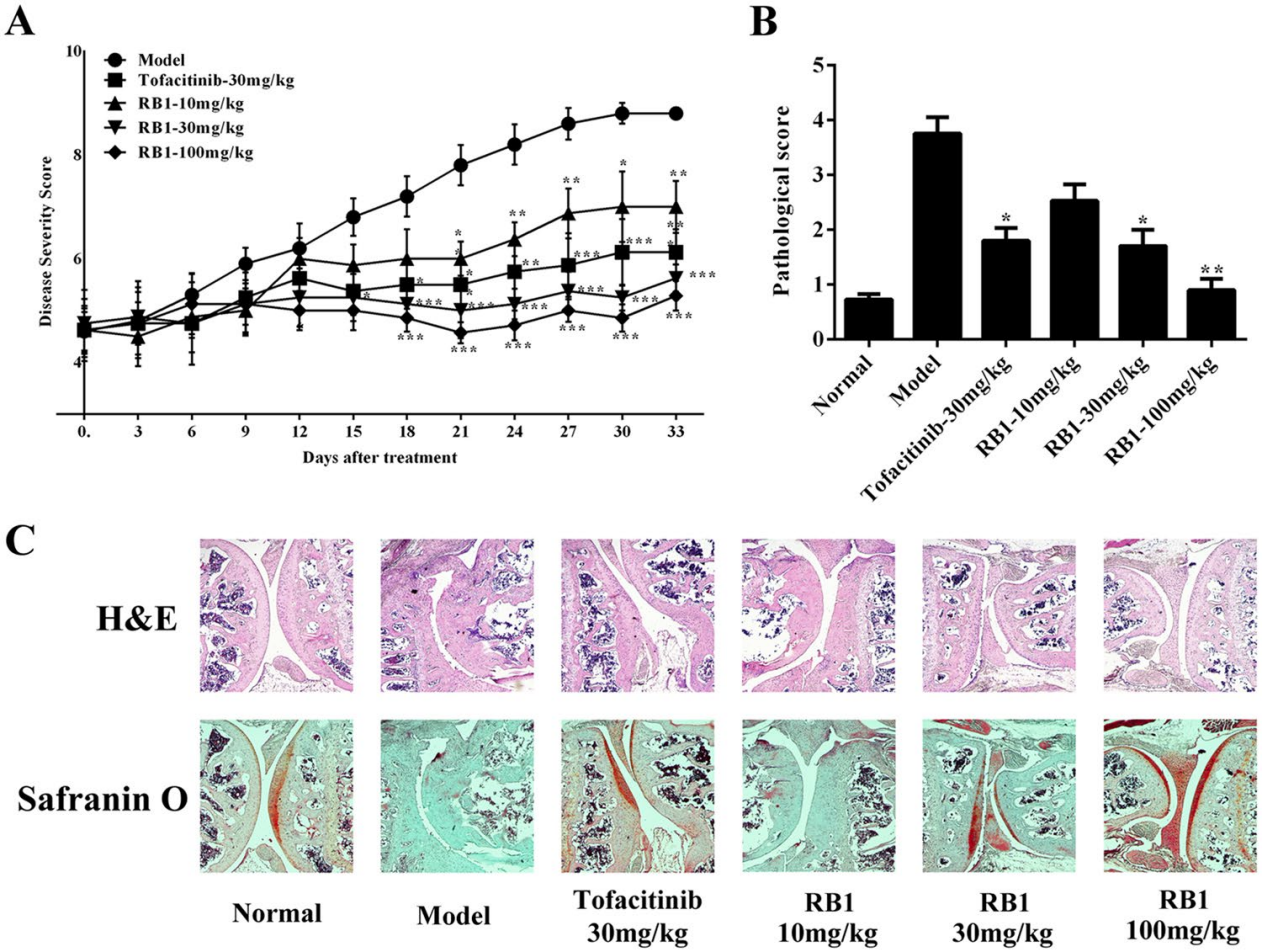
**RB1** is efficacious in treating a collagen-induced arthritis model. (**A**) Clinical scores of CIA mice after treatment with model, Tofacitinib (30 mg/kg), and **RB1** (10 mg/kg, 30 mg/kg or 100 mg/kg). Clinical scores were measured three days per time. (**B**) Pathological scores of the joint sections. Bars represent the mean ± S.E.M. (n = 10). *p < 0.05 and **p < 0.01 versus model. (**C**) H&E and safranin O-fast green staining of paraffin sections of ankle joints. Magnification =  ×40.

According to results above, we explored whether **RB1** could ameliorate the signs and symptoms of experimental arthritis^34^. Mice treated with **RB1** had lower arthritis scores than untreated CIA mice (p < 0.05 at 10 mg/kg and p < 0.001 at 30 and 60 mg/kg, Fig. 4A). The efficacy of **RB1** at 10, 30 and 100 mg/kg was comparable with that of Tofacitinib at 30 mg/kg^35^. Photos of representative joints and paw swelling in mice also exhibited the efficacy of **RB1** in treated mice (Supplementary Fig. S6A,B). The paws from model group mice had a group mean severity of 3.8, while the groups mean histological severity scores in the **RB1**-treated mice were 0.9, 1.7, and 2.5 at dosages of 100, 30, and 10 mg/kg, respectively. Meanwhile, the histological severity score in the Tofacitinib treated group was 1.8 (Fig. 4B). In H&E staining, joints from the model group mice showed severe inflammation of the synovium with a thickening of the lining layer, infiltration of inflammatory cells, and erosion of the cartilage and subchondral bone. In contrast, mice who were treated with **RB1**, especially at the doses of 30 and 100 mg/kg, showed fewer signs of inflammatory and cartilage and bone erosion. Cartilage destruction was further demonstrated by the safranin O staining. In the model group, the cartilage was extensively depleted of proteoglycan and devoid of chondrocytes, while treatment with **RB1** resulted in a dose-dependent reduction in the inflammation and damage to the articular cartilage (Fig. 4C). H&E analysis of the major organs indicated no significant side effects of the treatment; the body weight also showed no fluctuations during treatment with **RB1** (Supplementary Fig. S7A,B). These results suggested the **RB1** exhibited a better treatment capability than Tofacitinib in arthritis by relieving pathological processes.

### RB1 inhibits the JAK-STAT pathway in the collagen-induced arthritis model

**Figure 5:**
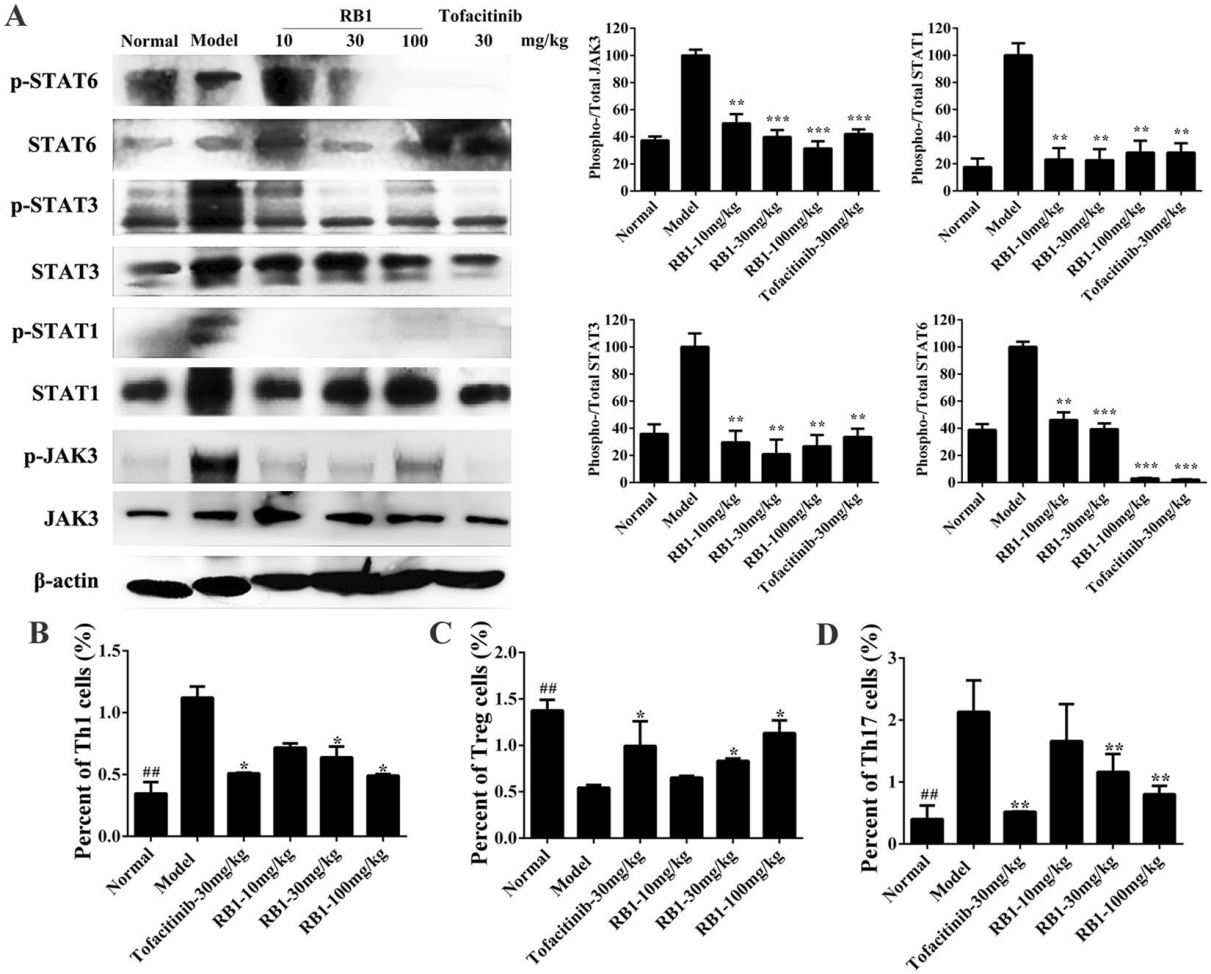
**RB1** inhibits the JAK-STAT pathway in a collagen-induced arthritis model. (**A**) The JAK/STAT signalling pathways in joints. Bars represent the mean ± S.E.M. (n = 3). (**B**) (**C**) (**D**) The statistical analyses of Th1, Treg and Th17 cells. Bars represent mean ± S.E.M. (n = 3). ^##^p < 0.01 versus normal to model, *p < 0.05, **p < 0.01 versus model. Uncropped images of blots are shown in Supplementary Fig. S11.

In CIA mice, the key signalling molecules involving the inflammatory process include the JAKs and the downstream effectors STATs. Hence, we examined the phosphorylation status of various JAKs and STATs in joint tissue from mice. **RB1** treatment of CIA mice resulted in hypo-phosphorylation of JAK3, STAT1 and STAT3 in a dose-dependent manners (Fig. 5A).

Proinflammatory cytokines play critical roles in the pathogenesis and progression of arthritis. Thus, we detected the production of TNF-α, IL-1β and IL-6 at the systemic level by ELISA. In the **RB1**-treated mice, serum TNF-α, IL-1β and IL-6 levels were significantly reduced in a dose-dependent manner (Supplementary Fig. S8B). As **RB1** reduced the proinflammatory cytokines levels in systemic plasma, we explored the efficiency of **RB1** in an inflammation-associated factor role in joint tissue. Using RT-PCR detection of inflammatory cytokine gene expression levels, we found significantly higher gene transcription levels of proinflammatory cytokines such as TNF-α, IFN-γ, IL-1, IL-2 and IL-6 from the articular tissue model group mice in comparison to the levels of normal mice (Supplementary Fig. S8A). In addition, treatment with **RB1** dose-dependently reduced the gene transcription levels of IL-1, IL-2 and IL-6. Similar results were also observed in the Tofacitinib-treated group. Interestingly, treatment with **RB1** can significantly reduced the TNF-α and IFN-γ gene transcription levels in joint tissue gene transcription (p < 0.01), but the reduced rate of IFN-γ was lower than that of the Tofacitinib treatment group. This result may involve the inhibition of JAK2 by Tofacitinib. As a highly selective inhibitor, **RB1** had less inhibition of JAK2; thus, the inhibition of IFN-γ was weaker than that of Tofacitinib *in vivo*. These results were consistent with the ELISA data. In addition, we observed that the levels of IL-10 was inhibited in the model group, and **RB1** and Tofacitinib significantly increased its expression (Supplementary Fig. S8A).

In CIA mice, T-cell differentiation into Th1 or Th17 is dependent on cytokines, while IL-6, TNF-α, and IL-1β can be suppressed via an IL-10-mediated feedback inhibition. Production of IL-10 can promote the generation of regulatory T cells (Treg cells) and inhibit the formation and differentiation of Th17 cells. As preliminary results showed that the expression of IL-10 was increased in the model mouse group, we explored the number of Th1, Th17 and Treg cells by flow cytometry analysis. In the analysis of Th1 cells, we found that Th1 cells were significantly increased (1.12%) in the model group in comparison to the normal group (0.35%), while **RB1** at 100 mg/kg could markedly inhibit the proliferation of Th1 cells (0.49%, p < 0.05) (Fig. 5B). The percentage of Treg cells in the model group was 0.55%, while in the 100 mg/kg group of **RB1** group, there was a significantly increased percentage of Treg cells (1.13%), which was much higher than that of the model group (p < 0.05) (Fig. 5C). In addition, compared with the model group (2.13%), **RB1** significantly reduced the percentage of Th17 cells at a dose of 100 mg/kg (0.80%, p < 0.01) (Fig. 5D, Supplementary Fig. S9). These results indicate that **RB1**, as a novel JAK3 selective inhibitor, has the potential to be a treatment for RA.

## Discussion

For many years, the front line treatment for RA has relied on the use of disease-modifying antirheumatic drugs (DMARDs), which are limited by side effects including nephrotoxicity and neurotoxicity due to the ubiquitous tissue distribution of their molecular targets^36,37^. In contrast, JAK3 is restricted in expression to immune cells^6,38^. Interestingly, JAK3 and STATs expression has been shown to be decreased in synovial tissue biopsies from patients with active RA receiving and responding to DMARDs therapy^39^. Therefore, a compound that can selectively inhibit JAK3 has theoretical advantages over existing immunosuppressive drugs. Tofacitinib, the pan-JAK inhibitor, provides a good treatment for RA but also brings several severe side effects, which are possibly caused by the inhibition of JAK1 and JAK2^12–14^. In this paper, we describe a newly discovered irreversible covalent JAK3 inhibitor **RB1**, which exhibits higher JAK isoform selectivity for JAK3 and shows suitable pharmacokinetics properties and low toxicity in pharmacodynamics evaluations.

As described above, we found that **RB1** shown an inhibition of JAK3 but not of the other JAKs. **RB1** selectively inhibited JAK3 with an over 100-fold preference above JAK2, JAK1, and TYK2 in the kinase assay. JAK3 is unique in having the cysteine residue Cys909 at the binding pocket, and this residue can be used to confer selectivity by the targeted formation of a covalent interaction^22–28^, but there are several other tyrosine kinases that carry a thiol in an analogous position to Cys909 in JAK3^40^, as described recently by a review summarizing all the work that has been performed in the field of covalent JAK inhibitors^27^. Our work confirmed that the potent JAK3-selective inhibition by **RB1** above other JAK isoforms can be achieved by capitalizing on the fact that JAK3 contains a unique Cys residue at position 909, allowing for the covalent irreversible inhibition of JAK3 (Fig. 3). The inhibition data from the Cys909-containing kinase assays shows favourable selectivity ratios for **RB1** compared to JAK3 (Table 2). Moreover, **RB1** displayed excellent kinase selectivity when tested against a panel of more than 70 kinases *in vitro* (Supplementary Table S1). Interestingly, five protein serine/threonine kinases, Aurora-A, Aurora-B, CLK2, MKK7β and PKG1α, were detected as also being inhibited by **RB1** to different degrees but less potently than JAK3, as the IC_50_ values of these kinases were all over 800 nM, indicating that **RB1** did not show favourable binding affinities to any the tested kinases except JAK3.

In the cellular setting, the decreased potency of **RB1** for JAK1 or JAK2 relative to JAK3 in the kinase assays resulted in an over-400 fold reduced potency for IL-6- or GM-CSF-mediated signalling relative to IL-2 mediated signalling in hPBMCs, suggesting that **RB1** shows a highly selective inhibition of JAK3 (Table 3). Since JAK1 pairs with JAK3 in all γ-chain-receptor-mediated signalling pathways^31^, and JAK2 is the most closely related member of the JAK family to JAK3^41^, the JAK3 selective inhibition by **RB1** was also tested in a battery of cytokine-stimulated cell-based assays (Fig. 2). The results showed **RB1** had a high selectivity against for JAK1 and JAK2 in cellular assays. We noted that, compared with JAK1/JAK2-mediated STATs phosphorylation, the inhibition of JAK3 phosphorylation in the IL-4 cell-based assay in THP-1 cells was over 1000-folds increased, suggesting that JAK3 inhibition by **RB1** is effective at blocking downstream signalling. This selective inhibition may result from the covalent irreversible inhibition of JAK3 by **RB1**, as described above.

The animal pharmacology studies shown in this work demonstrate that **RB1** effectively reduced inflammation and its associated pathology in CIA mice. **RB1** also demonstrated an inhibition of physiological processes that have been shown to underlie RA disease progression in humans, including cartilage damage and bone resorption^42^. Importantly, with **RB1** administration, proinflammatory cytokines and JAK3 and STATs phosphorylation decreased in mice, suggesting that the inhibition of JAK3/STAT signalling was closely correlated with inflammation in RA and that **RB1** was efficacious in the mouse CIA model of RA.

In summary, we have identified a novel and selective JAK3 inhibitor **RB1**, which blocks *in vitro* JAK3 kinase and functional activity in various cell types. When administered to mice orally, **RB1** has been proven to mediate the JAK-STAT pathway and reduce the clinical and microscopic manifestations of paw damage in CIA mice. These results indicate that the inhibition of JAK3 alone is sufficient to treat CIA mice and that selective JAK3 inhibitors may become novel therapeutic agents for the treatment of RA and other immune-related diseases.

## Methods

### Chemistry

HR-MS data for intermediates. ^1^H NMR (400 MHz, DMSO-*d*_6_): δ 12.98 (s, 1 H), 8.20 (s, 1 H), 8.11 (s, 1 H), 6.94 (d, 1 H, J = 6.4 Hz), 5.16–5.04 (m, 2 H), 3.34 (s, 1 H), 3.23–3.05 (m, 2 H), 1.90–1.79 (m, 2 H), 1.50 (t, 2 H, J = 4.2 Hz), 1.39 (s, 9 H); MS (ESI), m/z: 317.4 [M−H]^−^.

NMR and HR-MS data for **RB1**. ^1^H NMR (400 MHz, DMSO-*d*_6_): δ 13.03 (s, 1 H), 8.19 (s, 1 H), 8.17 (d, 1 H, *J* = 7.6 Hz), 8.11 (s, 1 H), 6.27 (dd, 1 H, *J* = 10.0 Hz, *J* = 17.2 Hz), 6.12 (dd, 1 H, *J* = 2.0 Hz, *J* = 17.2 Hz), 5.61 (dd, 1 H, *J* = 2.0 Hz, *J* = 10.0 Hz), 5.21–4.85 (m, 2 H), 3.82 (s, 1 H), 3.27–3.11 (m, 2 H), 2.05–1.73 (m, 2 H), 1.62–1.45 (m, 1 H), 0.89–0.63 (m, 1 H). ^13^C NMR (101 MHz, DMSO-*d*_6_) *δ*: 163.95, 153.22, 151.67, 151.42, 138.04, 131.64, 125.32, 118.61, 45.72, 45.25, 30.36, 23.49, 8.55. MS (ESI), m/z: 273.2 [M + H]^+^

### Enzymatic Assays

The enzymatic activities against JAK1, JAK2, JAK3, and TYK2 were tested by a Caliper Mobility Shift Assay with an ATP concentration at Km (ChemPartner; Shanghai; China). The assay protocol guides are commercially available from ChemPartner. The activities against the Cys-containing kinase families were tested by EurofinsCerep (CEREP, Celle l’Evescault, France). The protocols are available from http://www.cerep.fr/Cerep/Users/index.asp. The enzymatic activities against 81 representative protein kinases were tested by the Eurofins Kinase Profiler screening platform (Eurofins Pharma Discovery Services UK Limited, Dundee, United Kingdom). The assay protocol guides can be visited at http://www.eurofins.com/discoveryservices. In this assay, S(35) = (number of nonmutated kinases with ctrl% < 35%)/(number of nonmutated kinases tested); S(10) = (number of nonmutated kinases with ctrl% < 10%)/(number of nonmutated kinases tested); and S(1) = (number of nonmutated kinases with ctrl% < 1%)/(number of nonmutated kinases tested).

### JAK Cellular Assays

Raw peripheral blood mononuclear cells (PBMCs; All Cells) were isolated from the buffy coats of healthy volunteers using density gradient centrifugation on Lymphoprep. Cells were cultured in complete RPMI 1640 medium (containing 10% foetal bovine serum, 100 mg/ml streptomycin and 100 U/ml penicillin) plus 10 μg/ml lectin phytohemagglutinin (PHA) for 3 days and then treated with either recombinant human IL-6 (400 ng/ml; R&D Systems), recombinant human IL-2 (100 ng/ml; R&D Systems), or recombinant human GM-CSF (50 ng/ml; Pepro-Tech) at 37 °C for 20 min. To terminate the stimulation, cells were fixed with Lyse/Fix Buffer and then incubated with 100% methanol for 30 minutes; cells were incubated with anti-pSTAT3 and anti-CD4 Abs, or anti-pSTAT5 and anti-CD4 Abs (all Abs were from BD Biosciences) at 4 °C overnight, washed twice with PBS, and analysed with an FACS Canto II flow cytometer.

### STAT6 phosphorylation induced by IL-4

THP-1 cells (ATCC TIB-202) were preincubated with the compound at 37 °C for 1 h, incubated with IL-4 (10 ng/ml) at 37 °C for 60 min, and processed for Western blotting analysis.

### STAT5 phosphorylation induced by IL-3

TF-1 cells were starved overnight in RPMI 1640 medium with 0.1% FBS, preincubated with the compound at 37 °C for 1 h, stimulated with IL-3 (30 ng/ml) at 37 °C for 20 min, and processed for Western blotting analysis.

### STAT3 phosphorylation induced by IL-6

TF-1 cells were cultured in media for 18 h without GM-CSF prior to being plated in 24-well plates. The compound was added to the cells, and incubated for 30 min. Cells were then stimulated by IL-6 (400 ng/mL) incubation for 30 min and processed for Western blotting analysis.

### STAT1 phosphorylation induced by IFN-α and IFN-γ

U2OS cells were preincubated with the compound at 37 °C for 1 h, treated with 30,000 U/ml IFN-αB2 or 20 ng/ml IFN-γ at 37 °C for 1 h, and processed for Western blotting analysis.

### STAT5 phosphorylation induced by EPO

HEL cells were preincubated with the compound at 37 °C for 1 h, treated with 1 U/ml EPO at 37 °C for 1 h, and processed for Western blotting analysis.

### STAT3 phosphorylation induced by G-CSF

HEL cells were preincubated with the compound at 37 °C for 1 h, treated with 100 ng/ml G-CSF at 37 °C for 1 h, and processed for Western blotting analysis.

### Western Blot

Ankle joints of CIA mice were removed after sacrificed and ground by a mortar in liquid nitrogen. Both cells and abrasive products were lysed in RIPA buffer containing with the protease inhibitor cocktail, PMSF. Protein concentrations in the clarified lysates were determined, and samples were standardized to equal concentrations. Protein samples were then subjected to 10% sodium dodecylsulfate-polyacrylamide gel electrophoresis and transferred onto a polyvinylidene difluoride membrane. The membrane was blocked in 5% non-fat milk, incubated with primary antibodies, and subsequently incubated with the appropriate horseradish peroxidase-conjugated secondary antibody. Proteins were visualized with enhanced chemiluminescence.

### Protein Digestion for Mapping

JAK3 (0.1 mg/ml, 10 μl in 50 mM MOPSO, pH 6.5, 10 mM MgCl_2_, 2 mM MnCl_2_, 2.5 mM DTT, 0.01% BSA, 0.1 mM Na_3_VO_4_, 5% DMSO buffer) in the presence and absence of **RB1** was alkylated with 20 mM IAA for 45 min at room temperature in darkness, then reduced with 10 mM DTT for 1 h at 56 °C and again alkylated with 20 mM IAA for 45 min at RT in darkness. Then, trypsin was added at a 1:50 trypsin-to-protein mass ratio for the first digestion overnight and a 1:100 trypsin-to-protein mass ratio for a second 4-h digestion. The digested peptides were dried with a SpeedVac (Thermo) for LC-MS/MS analysis.

### UPLC-MS/MS Acquisition for Mapping

Peptides were dissolved in solvent A (0.1% FA in 2% ACN) and directly loaded onto a reversed-phase pre-column (Acclaim PepMap 100, Thermo Scientific). Peptide separation was performed using a reversed-phase analytical column (Acclaim PepMap RSLC, Thermo Scientific) with a linear gradient of 8–35% solvent B (0.1% FA in 98% ACN) for 12 min and 35–80% solvent B for 4 min at a constant flow rate of 320 nl/min on an EASY-nLC 1000 UPLC system. The resulting peptides were analysed by Q Exactive^TM^ Plus hybrid Quadrupole-Orbitrap Mass Spectrometer (ThermoFisher Scientific).

The peptides were subjected to NSI source followed by tandem mass spectrometry (MS/MS) in Q Exactive^TM^ Plus (Thermo) coupled online to the UPLC. Intact peptides were detected in the Orbitrap at a resolution of 70,000. Peptides were selected for MS/MS using 30% NCE; ion fragments were detected in the Orbitrap at a resolution of 17,500. A data-dependent procedure that alternated between one MS scan followed by 10 MS/MS scans was applied for the top 20 precursor ions above a threshold ion count of 5000 in the MS survey scan with 5.0 s dynamic exclusion. The electrospray voltage applied was 2.0 kV. Automatic gain control (AGC) was used to prevent overfilling of the ion trap; 5 E4 ions were accumulated for generation of MS/MS spectra. For MS scans, the m/z scan range was 350 to 1800.

### Reversibility assay

This test trial of our compound **RB1** was performed on a ChemPartner reversibility assay platform (ChemPartner; Shanghai; China). Jump dilution analysis is commonly used. The compound was tested in duplicate under 2 conditions, preincubation with enzyme and no preincubation. The assay protocol guide is commercially available from ChemPartner.

### Pharmacokinetics in Sprague Dawley rats

Two groups (n = 5) of male and female Sprague Dawley rats were fasted overnight and received **RB1** as an intravenous dose (5 mg/kg) or by oral gavage (10 mg/kg). Blood samples (0.4 mL) were obtained from retro-orbital bleeding at 5 min, 15 min, 30 min, 1 h, 2 h, 4 h, 6 h, 8 h, 10 h, 12 h, and 24 h post-dose for the p.o. group. At each time point, three rats were bled resulting in a composite pharmacokinetic profile. The sample tubes were inverted several times to ensure mixing and the placed on ice. The blood samples were centrifuged to obtain the plasma fraction. The plasma samples were deproteinized with acetonitrile containing aninternal standard. After centrifugation, the supernatant was diluted and centrifuged again. The compound concentrations in the supernatant were measured by a high-performance liquid chromatography-tandem mass spectrometry (LC/MS/MS). The obtained data were processed by the software DAS 2.0.

### Acute Toxicity and Haematology analysis

Four groups (n = 6) of male and BALB/c mice (20–22 g) were fasted overnight. In an acute toxicity test, mice were their behavioural or physiological symptoms were observed for 14 days. Haematological parameters examined in this study included WBC count, red blood cell (RBC) count, haemoglobin (HGB), red blood cell specific volume (HCT), mean corpuscular volume (MCV), mean corpuscular haemoglobin (MCH), mean corpuscular haemoglobin concentration (MCHC), platelets (PLT), lymphocytes (LY) and monocytes (MO).

### Collagen-Induced Arthritis Mouse Model

The animal protocol was approved by the Animal Care and Use Committee of Sichuan University in China (IACUC number: 20100318). DBA/1 J mice (male, 6 weeks old) were obtained from Jackson Laboratory (Bar Harbor, ME). One day before the start of the experiment, CII solution (2 mg/ml) was prepared with 0.05 M acetic acid and stored at 4 °C. Just before the immunization, equal volumes of IFA and CII were mixed by a homogenizer in a precooled glass bottle in an ice water bath. Arthritis was induced in 8-week old male DBA/1 mice by immunizations with a 1:1 emulsion of bovine type II collagen (Chondrex) and Freund’s complete adjuvant (Sigma-Aldrich) on day 0 and with a 1:1 emulsion of bovine type II collagen in Freund’s incomplete adjuvant (Sigma-Aldrich) on day 21. Treatment was initiated when >20% of the mice demonstrated signs of the disease. On the day treatment was initiated, the mice were randomly assigned to controls (no collagen injection plus vehicle; n = 8), collagen plus vehicle (n = 8), collagen plus **RB1** at 10 mg/kg (n = 8), collagen plus **RB1** at 30 mg/kg (n = 8), collagen plus **RB1** at 100 mg/kg (n = 8) and collagen plus Tofacitinib at 30 mg/kg (n = 8) groups. Mice were scored daily for signs of arthritis for 33 days. CIA development was inspected three times per week, and the inflammation of the four paws was graded from 0 to 4 (grade 0, paws with no swelling or focal redness; grade 1, paws with swelling of finger joints; grade 2, paws with a mild swelling of the ankle or wrist joints; grade 3, paws with severe inflammation of the entire paw; and grade 4, paws with deformity or ankylosis). Each paw was graded, and the four scores were totalled so that the possible maximum score per mouse was 16.

### Histological examination

DBA1/J mice were sacrificed on day 33 after treatment and ankle joints were removed and fixed with 4% paraformaldehyde for more than 48 h. The joints were decalcified in EDTA buffer for 21 days and then embedded in paraffin blocks. Joint sections were stained with H&E or safranin O-fast green. Histological changes were examined under microscope using methods modified from McKew *et al*.^43^ as follows: grade 0 = no abnormal findings; grade 1 = synoviocyte hypertrophy, slight synovial membrane fibrosis, slight-to-mild inflammatory cell infiltrates into the synovial membrane/articular capsule and/or joint space; grade 2 = grade 1 plus mild-to-moderate inflammatory cell infiltrates, pannus formation (if present) minimal with superficial cartilage erosion; grade 3 = grade 2 plus marked inflammatory cell infiltrates and fibrosis, mild-to-severe erosion of the cartilage extending into the subchondral bone; and grade 4 = loss of joint integrity through erosion or destruction with bone remodelling, massive inflammatory cell infiltrates, fibrosis, and ankylosis.

### Gene expression analysis in mouse paws

Hind paws were dissected by cutting above the ankle joint and removing the digits. Total RNA was extracted using a RNA extraction kit (Axygen, USA). Template cDNA was synthesized using the Primescript RT reagent kit with gDNA eraser (Takara, Tokyo, Japan). Real-time quantitative PCR was performed using the following primers:

GAPDH-Forward primer: 5′-GTATGACTCCACTCACGGCAAA-3

GAPDH-Reverse primer: 5′-GGTCTCGCTCCTGGAAGATG-3′

IL-1β-Forward primer: 5′-TGGGCCTCAAAGGAAAGAAT-3′

IL-1β-Reverse primer: 5′-CAGGCTTGTGCTCTGCTTGT-3′

IL-2-Forward primer: 5′-AGCTCGCATCCTGTGTCAC-3′

IL-2-Reverse primer: 5′-TGACAGAAGGCTATCCATC-3′

IL-6-Forward primer: 5′-ACAACCACGGCCTTCCCTACTT-3′

IL-6-Reverse primer: 5′-CACGATTTCCCAGAGAACATGTG-3′

IL-10-Forward primer: 5′-GCTGGACAACATACTGCTAAC-3′

IL-10-Reverse primer: 5′-GCAGTTGATGAAGATGTC-3′

TNF-α-Forward primer: 5′-ACCCTCACACTCAGATCATCTTC-3′

TNF-α-Reverse primer: 5′-TGGTGGTTTGCTACGACGT-3′

IFN-γ-Forward primer: 5′-CACTGCATCTTGGCTTTGC-3′

IFN-γ-Reverse primer: 5′-AACAGCTGGTGGACCACTC-3′

### Flow cytometry analysis of Th1, Th17 and Treg in CIA mice

On day 32 after the indicated treatments, mice were sacrificed to obtain cells from the spleen. Then, the cells were incubated with the corresponding antibodies to detect Th1, Th17 cells and Treg cells. CD4^+^ IFN-γ^+^ represents Th1 cells, CD4^+^ IL-17^+^ represents Th17 cells in total joint cells. CD4^+^ Foxp3^+^ represents Treg cells. The analyses of cells were performed as described above.

### Statistical analysis

Statistics results of multiple experiments are expressed as the mean ± S.E.M. Student’s t-tests and one-way ANOVAs test were employed for data analysis. Significance was determined at P values of < 0.05.

### Ethics statement

All the methods were carried out in accordance with the approved guidelines, and the full experimental procedures were carried out under the guidance of the Institutional Animal Care and Use Committee of Sichuan University (Chengdu, China). Informed consent for the use of human normal peripheral blood samples for the present study was obtained from all patients according to the Declaration of Helsinki.

## Electronic supplementary material

Supplementary information
