# Supplementary material for: Discovery of a highly selective JAK3 inhibitor for the treatment of rheumatoid arthritis
Source: Sci Rep. 2018 Mar 27;8:5273. doi: 10.1038/s41598-018-23569-y (PMC5869712; doi:10.1038/s41598-018-23569-y)
Supplement: Supplementary file 1 — Supplementary information [file 41598_2018_23569_MOESM1_ESM.pdf]

SUPPLEMENTARY INFORMATION:

Discovery of a highly selective JAK3 inhibitor for the treatment of rheumatoid arthritis

Heying Pei[a,†], Linhong He[a,†], Mingfeng Shao[a,†], Zhuang Yang[a], Yan Ran[a], Dan Li[a], Yuanyuan Zhou[a], Minghai Tang[a], Taijin Wang[a], Yanqiu Gong[a], Xiaoxin Chen[b], Shengyong Yang[a], Mingli Xiang[a,b*], and Lijuan Chen[a,b*]

[a]State Key Laboratory of Biotherapy/Collaborative Innovation Center of Biotherapy and Cancer Center, West China Hospital of Sichuan University, Chengdu, China

[b]Guangdong Zhongsheng Pharmaceutical Co., Ltd., Dongguan, Guangdong 523325, China.

[†]These authors contributed equally to this work.

*Address correspondence to: Lijuan Chen, State Key Laboratory of Biotherapy and Cancer Center, West China Hospital of Sichuan University, Chengdu, China; Email: chenlijuan125@163.com

Additional correspondence to: Mingli Xiang, State Key Laboratory of Biotherapy and Cancer Center, West China Hospital of Sichuan University, Chengdu, China.

Supplementary Table S1. Kinome Selectivity Profiles of RB1[a]

Kinase	%	Kinase	%	Kinase	%
Abl	**112**	GRK5	**94**	Pim-2	**98**
ACK1	**34**	GSK3α	**82**	PKA	**85**
ALK	**100**	GSK3β	**114**	PKBα	**94**
ALK4	**101**	IGF-1R	**93**	PKCθ	**80**
Aurora-A	**21**	IKKα	**97**	PKCζ	**106**
Aurora-B	**12**	IKKβ	**105**	PKG1α	**23**
B-Raf	**90**	IKKε	**97**	Plk3	**99**
CaMKI	**96**	IR	**114**	PRK2	**79**
CaMKIIβ	**85**	JAK3	**0.9**	Ret	**86**
CaMKK2	**101**	JNK1α1	**101**	ROCK-I	**88**
CDK1	**96**	JNK2α2	**109**	ROCK-II	**88**
CDK2	**93**	KDR	**96**	Rsk2	**76**
CDK6	**100**	Lck	**80**	SAPK2a	**100**
CDK7	**95**	Lyn	**118**	SAPK2b	**103**
CDK9	**101**	MAPK1	**85**	SAPK3	**94**
CK1	**82**	MAPKAP-K2	**97**	SAPK4	**95**
CLK2	**18**	MEK1	**92**	SGK	**92**
DDR1	**75**	Mer	**68**	Src	**102**
DYRK1A	**91**	MKK4	**114**	STK33	**93**

DYRK1B	**86**	MKK6	**69**	Syk	**104**
FAK	**72**	MKK7β	**20**	TAO1	**70**
FGFR1	**80**	MSK1	**74**	TBK1	**96**
Flt1	**93**	MST1	**83**	TrkA	**46**
Flt3	**75**	NEK2	**99**	TrkB	**38**
Fms	**90**	PAK4	**81**	TrkC	**75**
Fyn	**97**	PDGFRα	**96**	Wee1	**90**
GCK	**80**	Pim-1	**62**	ZIPK	**107**

[a]Values represent percent inhibition at 1 μM concentration, data are the mean of at least n= 2 independent measurements. Lower numbers indicate stronger binding, where Negative control= DMSO (% inhibition= 100%)

.

Supplementary Table S2. Potency and selectivity of RB1 in cellular assays

				IC_{50} (nM)			Selectivity	
JAKs Involved	Cell Type	Trigger	Readout	JAK1	JAK2	JAK3	JAK1/JAK3	JAK2/JAK3
				RB1			RB1	
JAK1/JAK3	THP-1	IL-4	pSTAT6	>50000	-	53.1	>1000	-
JAK1	TF-1	IL-6	pSTAT3	>50000	-	-	>1000	-
JAK1	U2OS	IFN-α2B	pSTAT1	>50000	-	-	>1000	-
JAK2	TF-1	IL-3	pSTAT5	-	>50000	-	-	>1000
JAK2	TF-1	GM-CSF	pSTAT5	-	>50000	-	-	>1000
JAK1/JAK2	U2OS	IFN-γ	pSTAT1	>50000	>50000	-	>1000	>1000
JAK2	HEL	EPO	pSTAT5	-	>50000	-	-	>1000
JAK1/JAK2	HEL	G-CSF	pSTAT3	>50000	>50000	-	>1000	>1000

Supplementary Table S3. Pharmacokinetics profiles of RB1 in Rats[a]

	**iv**	**po**
**AUC_{0-t}** (μg/L*h)	**801.97**	**1163.17**
**$AUC_{0-\infty}$** (μg/L*h)	**933.02**	**1311.77**
**$T_{1/2}$** (h)	**14.60**	**6.90**
**T_{max}** (h)	**0.08**	**0.25**
**CL** (L/h/kg)	**5.86**	**8.65**
**V** (L/kg)	**98.04**	**77.33**
**C_{max}** (μg/L)	**1089.47**	**308.70**
**F %**	**—**	**72.52**

[a]Pharmacokinetics measured by analysis of plasma concentrations at indicated time points. Data were mean concentrations in plasma (n= 5) following a single 5.0 mg/kg intravenous dose or 10 mg/kg oral dose.

Supplementary Table S4. Hematology analysis of acute toxicity study of RB1 via oral administration in Balb/c mice.

	Normal	RB1<br>100 mg/kg	RB1<br>300 mg/kg	RB1<br>1000 mg/kg
WBC (1×10^9/L)	9.36 ± 1.35	9.12 ± 0.98	8.64 ± 1.05	9.33 ± 1.12
RBC (1×10^{12}/L)	9.85 ± 0.19	9.20 ± 0.31	9.90 ± 0.22	10.44 ± 0.16
HGB (g/L)	163.30 ± 7.01	168.12 ± 7.36	158.49 ± 6.40	150.33 ± 6.94
HCT (%)	47.32 ± 1.34	46.94 ± 1.80	44.82 ± 1.77	43.53 ± 1.68
MCV (fL)	49.97 ± 0.11	48.98 ± 0.22	50.03 ± 0.08	49.82 ± 0.12
MCH (pg)	16.10 ± 0.14	16.13 ± 0.12	15.98 ± 0.22	16.08 ± 0.09
MCHC (g/L)	363.26 ± 2.95	369.77 ± 2.01	360.54 ± 3.84	355.00 ± 2.16
PLT (1×10^9/L)	379.46 ± 29.01	385.34 ± 43.61	360.12 ± 33.74	327.67 ± 46.45
LY (%)	72.45 ± 2.09	65.93 ± 3.09	75.04 ± 3.17	69.43 ± 1.88
MO (%)	9.01 ± 0.77	6.99 ± 1.26	6.50 ± 0.86	8.23 ± 1.08

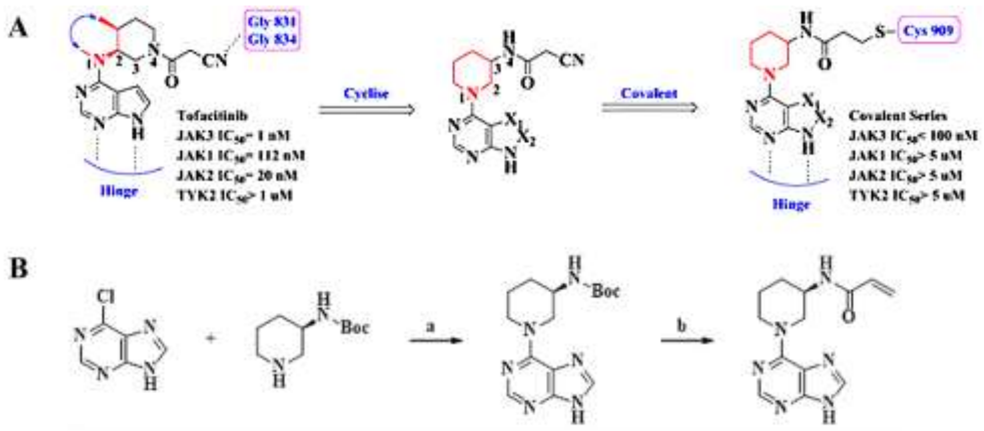

Supplementary Figure S1. The design strategy and Synthesis of RB1. (A) The design strategy of compound. (B) Synthesis of RB. Reagents and conditions: (a) pyridine, rt; (b) (i) TFA/DCM = 1:1, rt; (ii) acryloyl chloride, Et_3N, THF, rt;

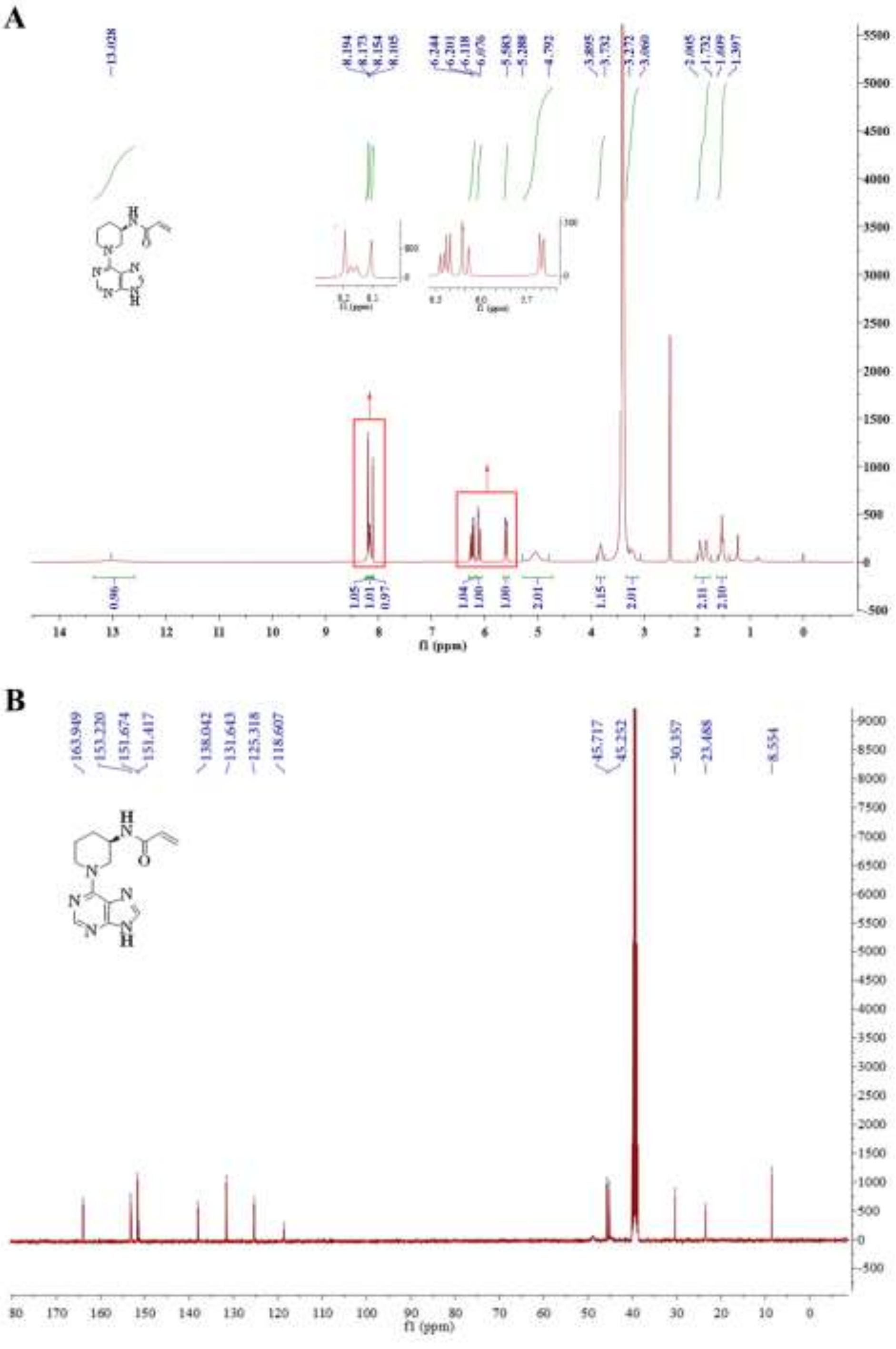

Supplementary Figure S2. The 1H-NMR and 13C-NMR data of RB1.

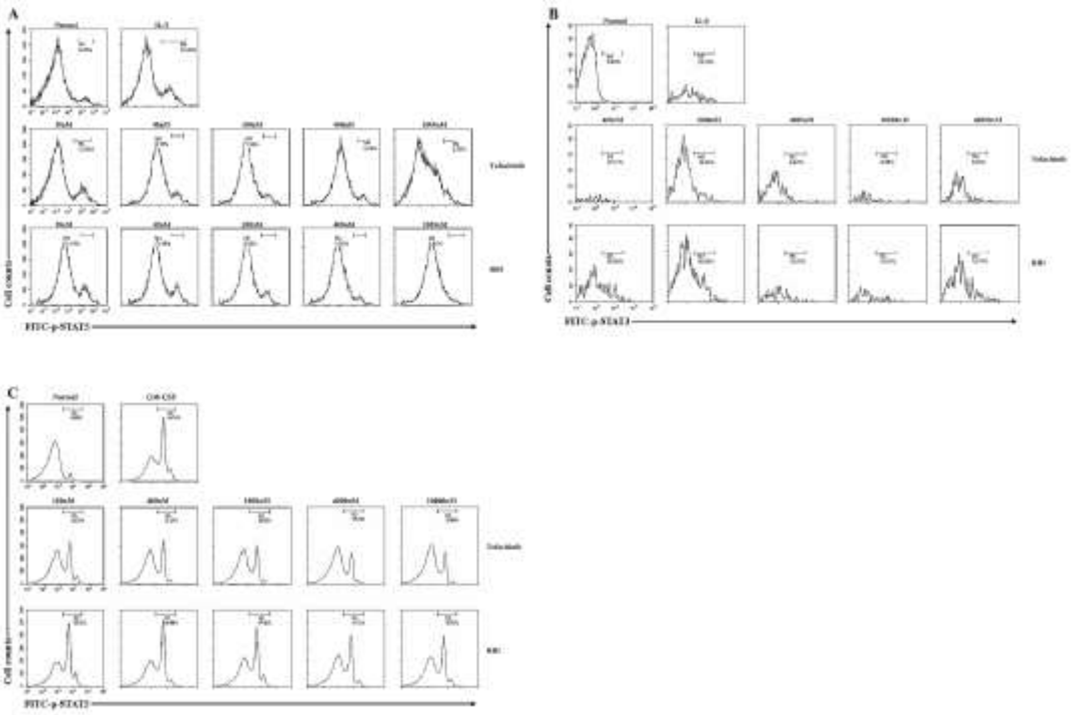

Supplementary Figure S3. The histogram data of flowcytometry after cytokines stimulation in the presence of RB1.

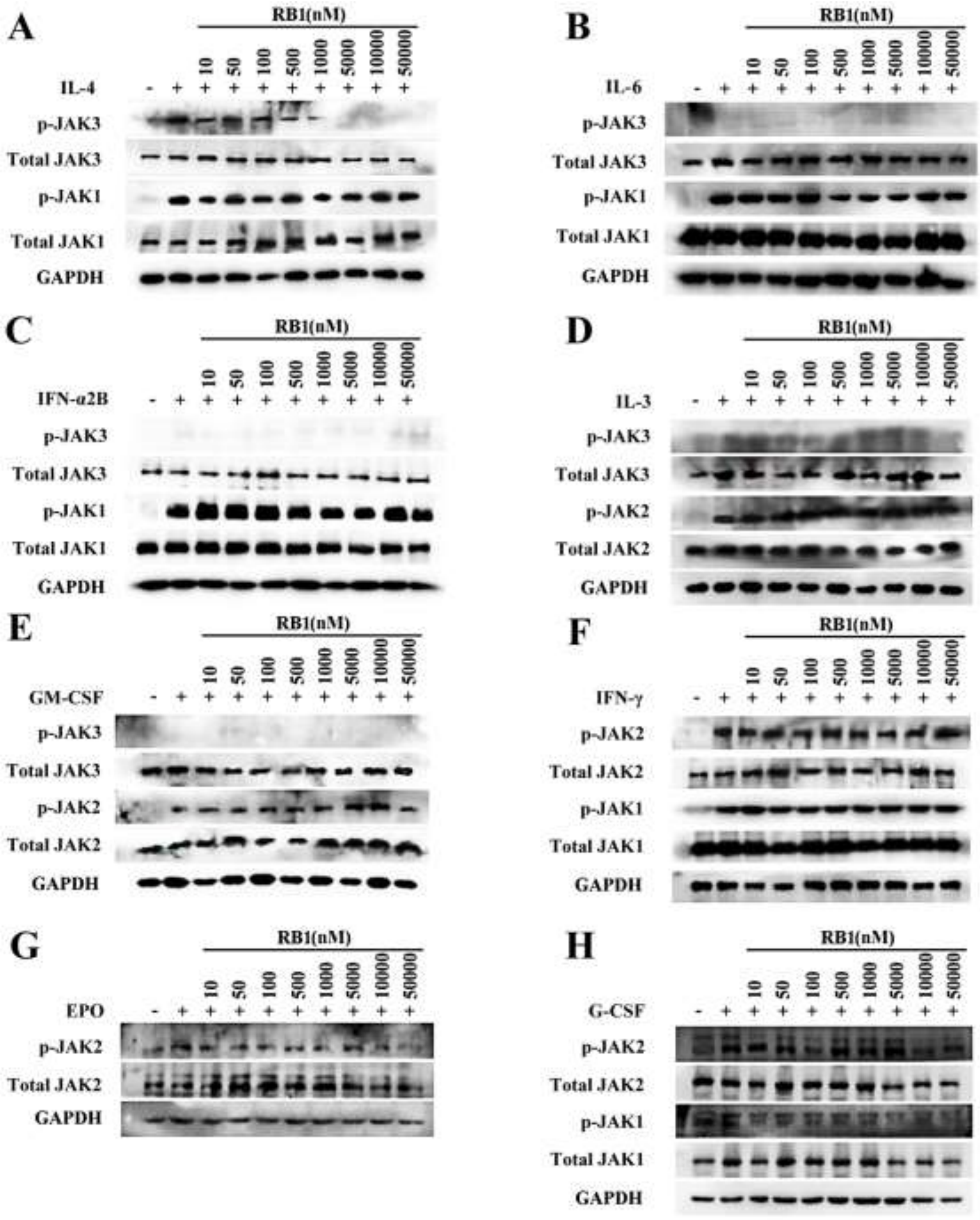

Supplementary Figure S4. Inhibitions of cytokine induced JAK auto-phosphorylation by RB1 in THP-1 cells (A), TF-1 cells (B), U2OS cells (C), TF-1 cells (D), TF-1 cells (E), U2OS cells (F), HEL cells (G), HEL cells (H). Uncropped images of blots are shown in Supplementary Figure S12.

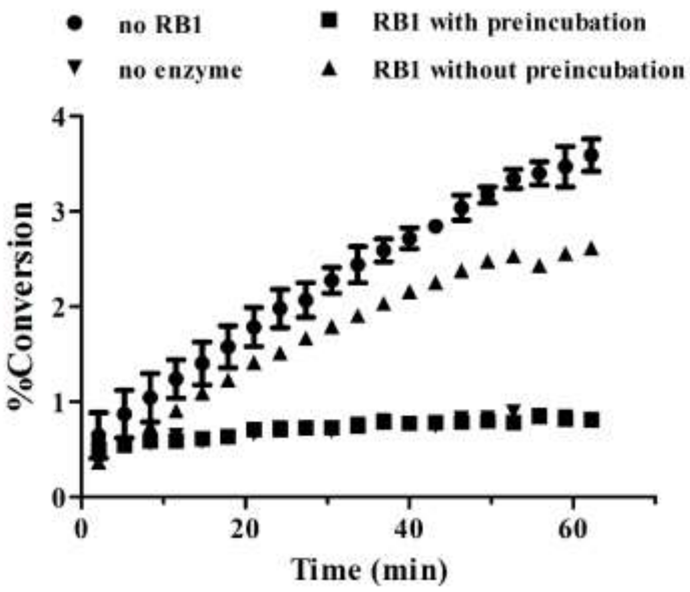

Supplementary Figure S5. RB1 irreversibly inhibited JAK3. RB1 with pre-incubation: compound RB1 was pre-incubated with AK3 for 30 min, then diluted with kinase buffer that contains peptide substrate, ATP and cofactors at the final concentration for reaction, and read conversion data for 20 times for 1 h on caliper.

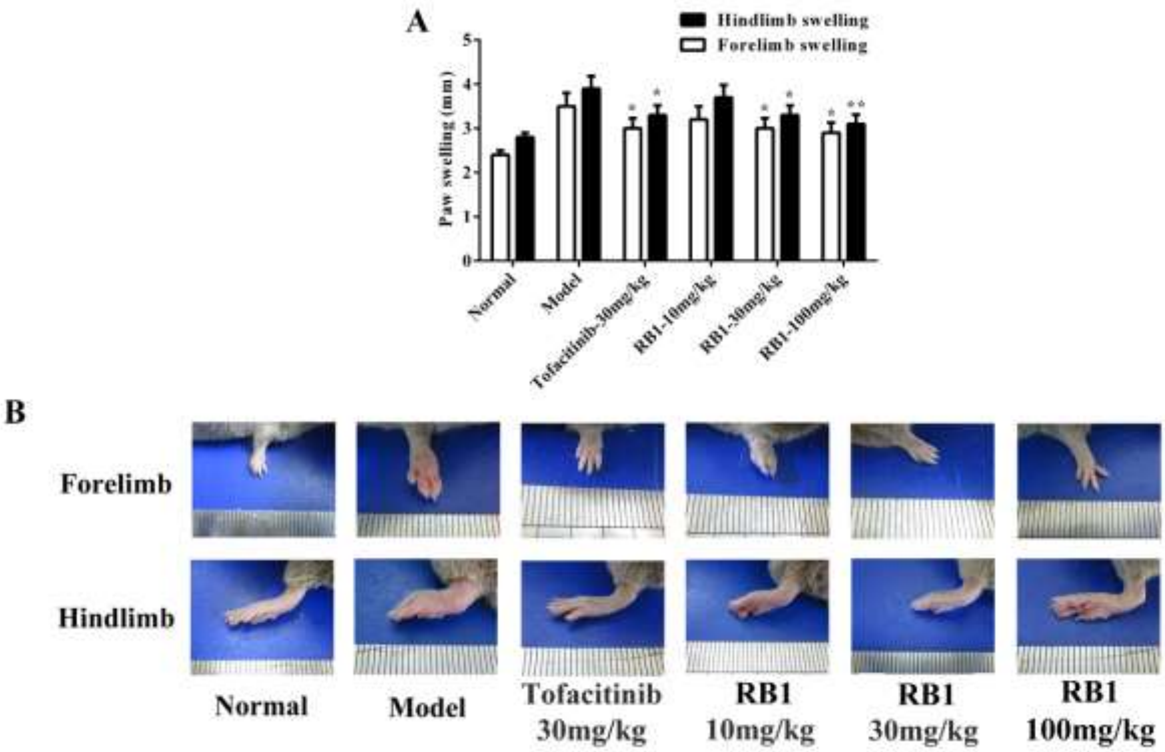

Supplementary Figure S6. Paw swelling of mice after treated, related to Figure 4. (A) paw swelling of CIA mice. On day 32 after treatment, paw swelling of CIA mice was measured with a slide gauge. Bars represent mean ± S.E.M. (n=10). * P <0.05, ** <0.01 versus Model. (B) Photos of representative joints on day 32 after treatment.

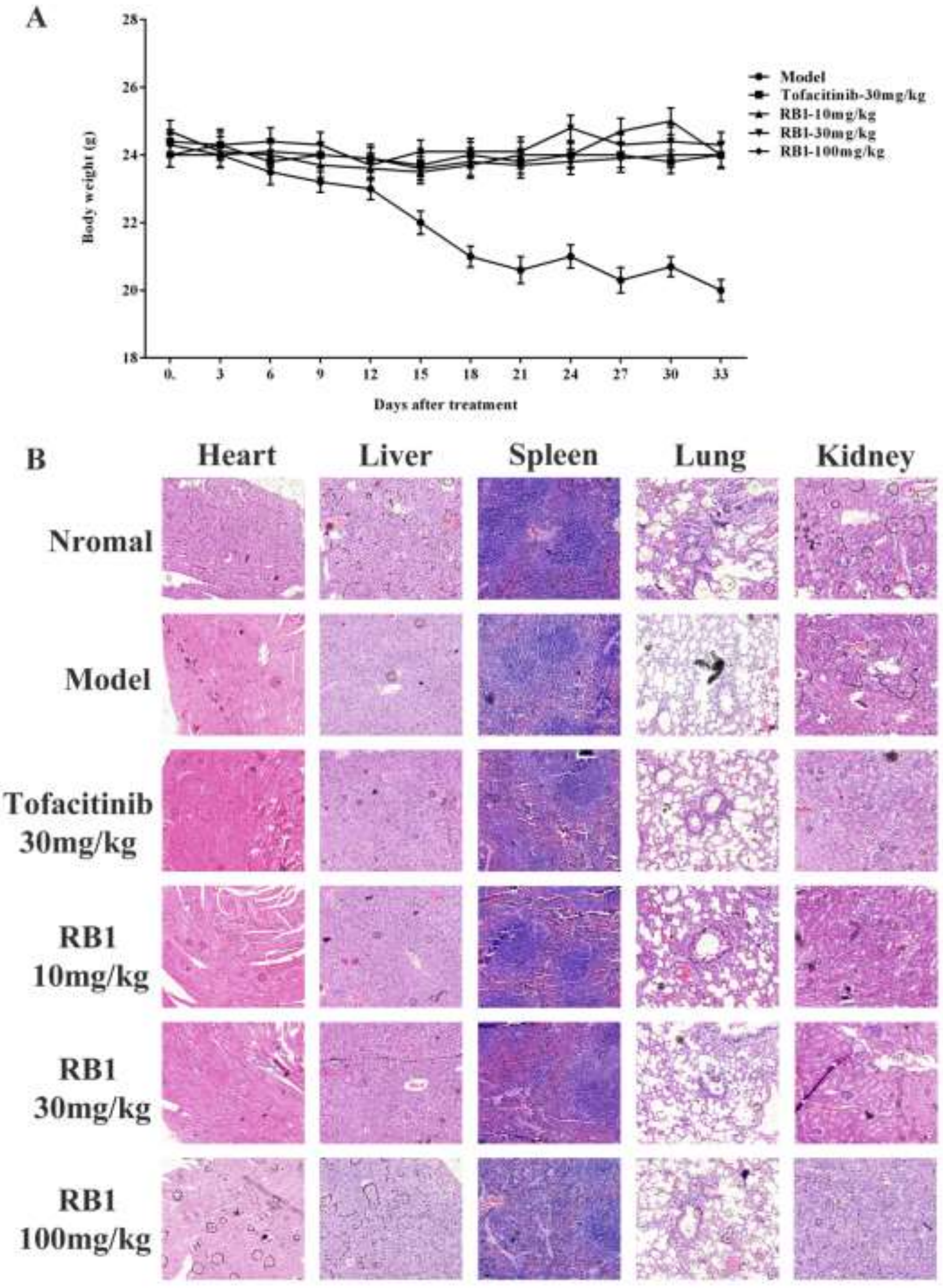

Supplementary Figure S7. Safety analysis of RB1. (A) Body weight of CIA mice. Bars represent mean ± S.E.M. (n=10). (B) HE analysis of major organs (heart, liver, spleen, lung, and kidney) in mice.

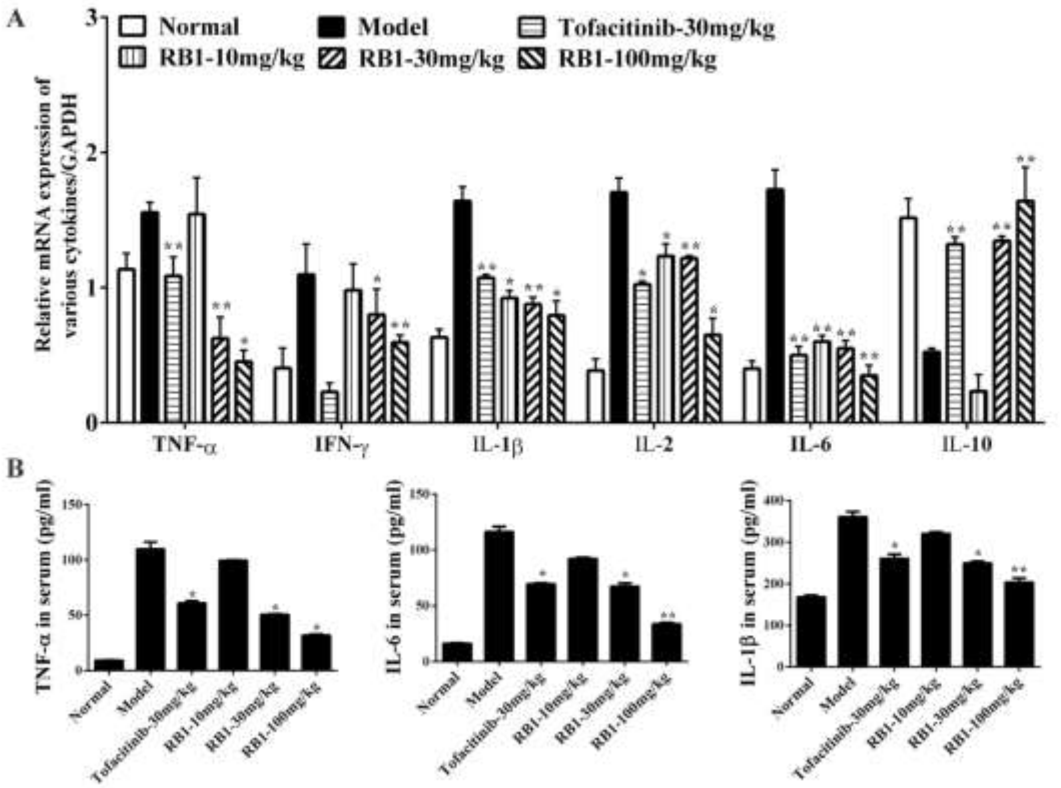

Supplementary Figure S8. Detection of gene and serum levels in CIA mice. (A) Gene levels of cytokines in joints by RT-PCR. On day 32 after indicated treatments, Total RNA was extracted from representative joints of CIA mice. Then the gene levels of proinflammatory and anti-inflammatory cytokines were detected by semiquantitative RT-PCR, and GAPDH was used as control. (B) The effect of RB1 on level of serum cytokines (TNF-α, IL-6, IL-1β) in CIA mice. Bars represent mean± S.E.M. (n=10). * P <0.05, ** P < 0.01 versus Model.

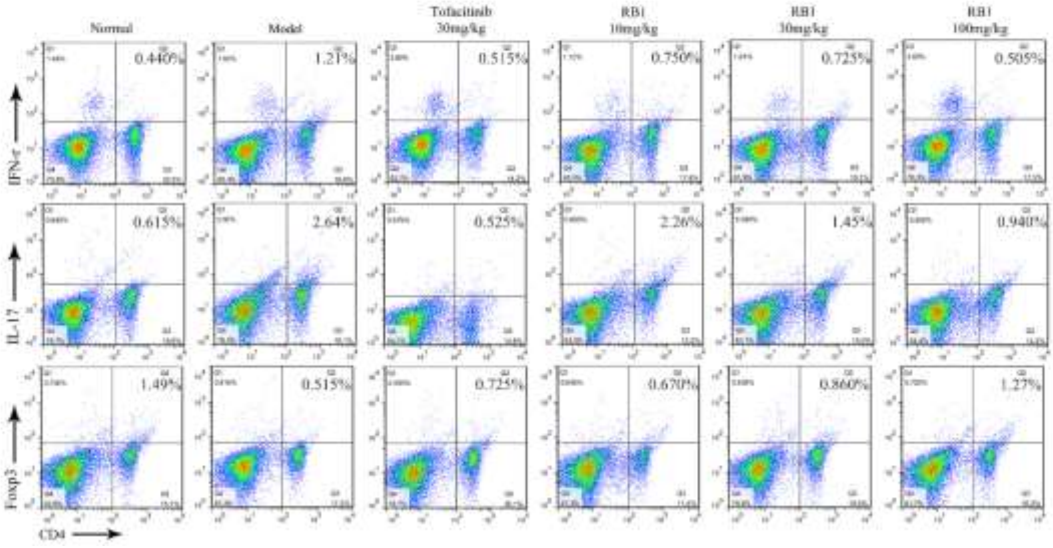

Supplementary Figure S9. The dot plot scattergrams of Th1, Th17 and Treg cells. On day 32 after the indicated treatments, mice were sacrificed to obtain cells in spleen. Then the cells were incubated with corresponding antibodies to detect Th1, Th17, Treg cells.

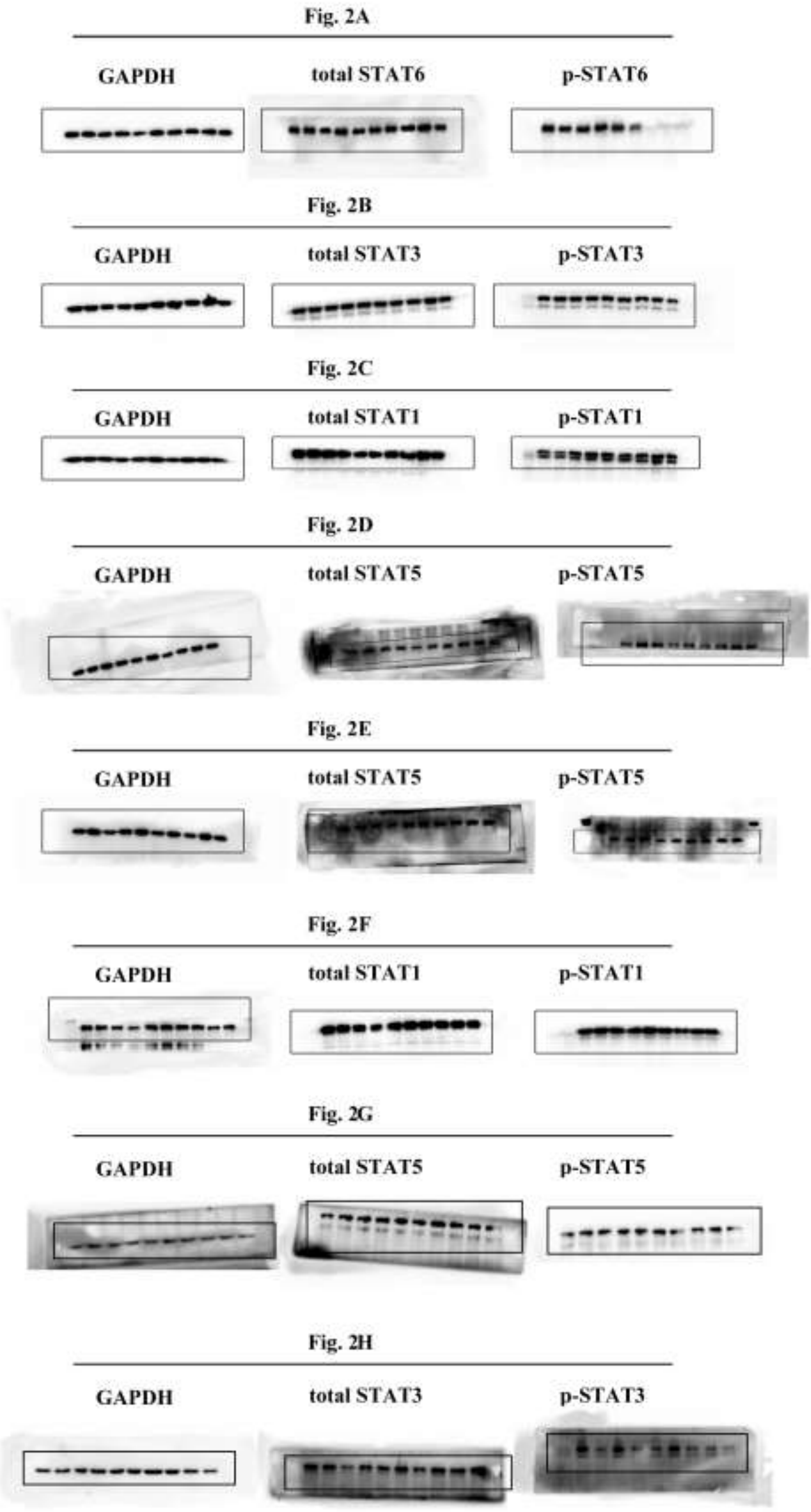

Supplementary Figure S10. Complete western blotting gels from Fig. 2.

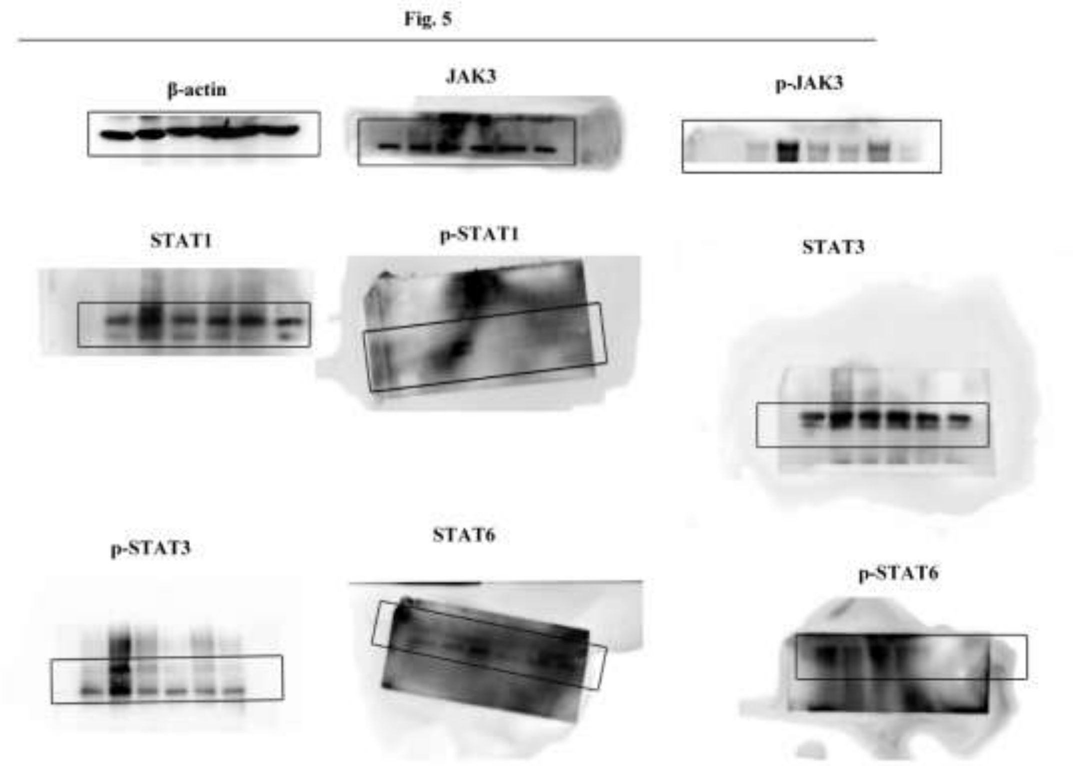

Supplementary Figure S11: Complete western blotting gels from Fig. 5.

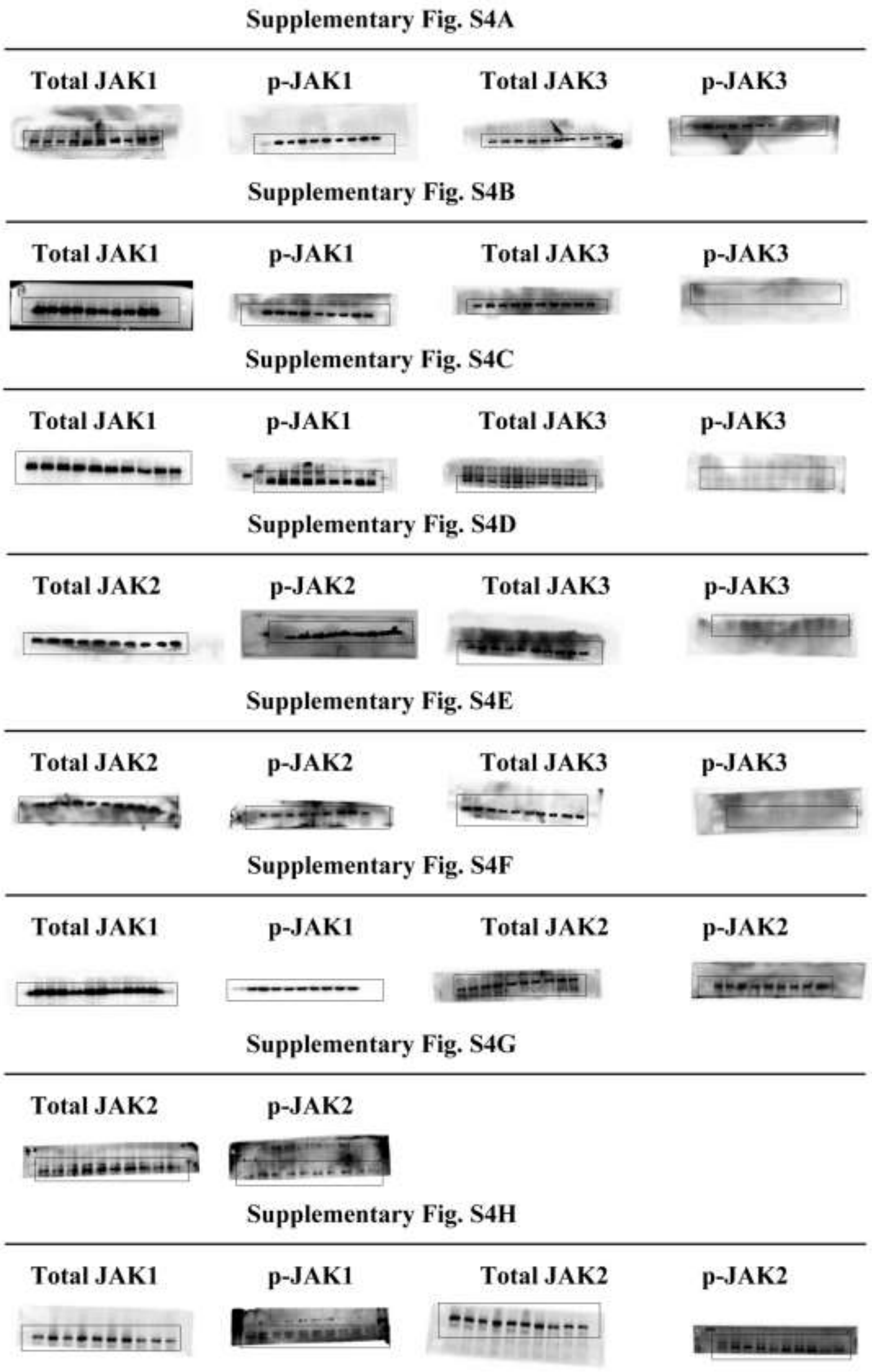

Supplementary Figure S12. Complete western blotting gels from Supplementary Fig. S4.
